# Organotypic Culture of Adult Vascularized Porcine Retina Explants In Vitro on Nanotube Scaffolds

**DOI:** 10.1186/s12575-025-00301-5

**Published:** 2025-09-08

**Authors:** Sabrina Friebe, Solveig Weigel, Mike Francke, Stefan G. Mayr

**Affiliations:** 1 Division of Surface Physics, Department of Physics and Earth System Sciences, University of Leipzig, Linnéstr. 5, 04103 Leipzig, Germany; 2 Department of Biocompatible and Bioactive Surfaces, Leibniz Institute of Surface Engineering (IOM), Permoserstr. 15, 04318 Leipzig, Germany; 3 Medical Informatics Center, University Hospital Leipzig, Härtelstr. 16-18, 04107 Leipzig, Germany; 4 Paul-Flechsig-Institute of Brain Research, University of Leipzig, Liebigstr. 19, 04103 Leipzig, Germany; 5 University Clinic and Polyclinic for Ophthalmology, University Hospital Halle (Saale), Ernst-Grube-Str. 40, 06120 Halle (Saale), Germany

**Keywords:** Long-term organotypic tissue culture, Vascularized retina, Retina explants, Assay for retina culture, Nanotube scaffolds

## Abstract

**Background:**

Organotypic long-term cultivation of vascularized retina explants is a major challenge for application in drug development, drug screening, diagnostics and future personalized medicine. With this background, an assay and protocol for organotypic culture of vascularized retina explants in vitro with optimum tissue integrity preservation is developed and demonstrated.

**Methods:**

Morphological, histologic and biochemical integrity as well as viability of vascularized retina explants are compared as function of cultivation time for differently structured nanotube scaffolds. In doing so, porcine retina explants obtained from a local slaughterhouse are employed as paradigm for vascularized retina.

**Conclusions:**

We demonstrate that titania nanotube arrays are highly promising as culturing scaffold of vascularized retina explants in vitro due to highly tunable surface properties regarding biomedical signaling. The unprecedented maintenance of tissue integrity allows for screening of pharmacological drugs and disease mechanisms in an ex-vivo test-based culture system with reduced need for animal experiments.

**Supplementary Information:**

The online version contains supplementary material available at 10.1186/s12575-025-00301-5.

## Introduction

Functionality, structure and elastic properties of the eye and certain parts of it, including the retina, have long been the subject of research [1–5]. The retina is constantly exposed to various mechanical stresses, for instance, compressive forces from the vitreous body [6]. Several diseases such as the age-related macular degeneration, retinal detachment, glaucoma or even blunt trauma injuries and their treatment have been the focus of many studies and have been shown to be associated with structural and mechanical properties changes [7–14]. However, many questions remain unanswered. While there are many studies on the mechanical properties of single cells of the retina, vitreous body, choroid or sclera, there are only few studies on the holistic retinal tissue, due to its high sensitivity and handling difficulties caused by its thinness and softness [1–3, 7, 15–17]. At the same time, it is difficult to test drugs for healing in adult retinal tissue in vitro, because the retina is very demanding and degenerates if cultured incorrectly, resulting in a loss of structure and changes in mechanical properties [13, 14]. However, long-term culture of adult neuronal tissue pose a significant challenge to a lack of regenerative capacity [18]. Some biological mechanisms or mechanical responses, for example in preclinical drug testing, can only be performed on fully differentiated tissues. Hence, organotypic cultures of primary tissue explants play an important role as a bridge between in vitro cell culture studies and in vivo experiments. Therefore, it would be of great interest and urgent need to develop a sensitive model system that allows long-term organotypic culture of vascularized tissue for drug delivery, studies of healing processes, or examinations of biomechanical properties, avoiding an animal in vivo-test based approach.

While previous studies, including our own work, have demonstrated the feasibility of using TiO_2_ nanotube scaffolds for organotypic culture of neuronal tissue and avascular retina explants, several critical challenges remain unaddressed [19, 20]. Most notably, there is a lack of robust, reproducible culture systems for adult, vascularized retina, which is structurally and functionally closer to the human retina and essential for transitional research and drug screening. The present study was therefore designed to systematically adapt and evaluate nanotube scaffolds for the long-term culture of adult, vascularized porcine retina explants. By optimizing scaffold geometry and surface properties, we aim to maximize culture success, including tissue integrity, cell viability, and adhesion, enabling reliable ex vivo models for preclinical applications. Our approach offers several key advantages, such as reproducible and ethically favorable alternative to in vivo animal experiments, as porcine eyes from the slaughterhouse can be used in accordance with the 3R principles. Also, it bridges the gap between conventional and in vitro models and in vivo studies, allowing for investigation of biological mechanism, drug response and mechanical properties in adult tissue.

Currently, adult tissue culture is mostly performed using Polytetrafluoroethylene (PTFE) culture inserts [21]. However, they demonstrate the disadvantage that these culture inserts cannot be tailored for different tissue or cell types. Therefore, the direct application is limited to tissues or cells that can adapt to the cultivation membrane or to tissues that only require the surface properties of the inserts. TiO_2_ nanotube scaffolds are a tailorable culture system that allows organotypic preservation of avascular retinal tissue, as we have already demonstrated in previous studies [18, 19]. The customizable hexagonally aligned nanotopography well promotes complete tissue and extracellular matrix (ECM) adhesion. Surface characteristics such as superhydrophilicity or surface charge can be individually adapted. Typical nanotube diameter used in our lab ranges from 20 to 120 nm. We also demonstrated that they are reusable and highly suitable as a basis for mechanical measurements of the tissue cultured thereon [20, 22, 23]. In this study, we introduce nanotube scaffolds as an ex vivo culture system that enables organotypic culture (currently several days up to one week) of an adult vascularized porcine retina, as we preserve the tissue’s highly complex morphology without perfusion system. The vascularized retina is highly demanding, as the nutrient supply is not only ensured by diffusion, but additionally by blood vessels. These results will be of great interest as they may form the basis for successful transfer to other entities and benefit medical-related research or applications, such as long-term drug testing, investigation of tissue healing processes, or personalized medicine.

## Methods

### TiO_2_ Nanotube Synthesis

**Fig. 1:**
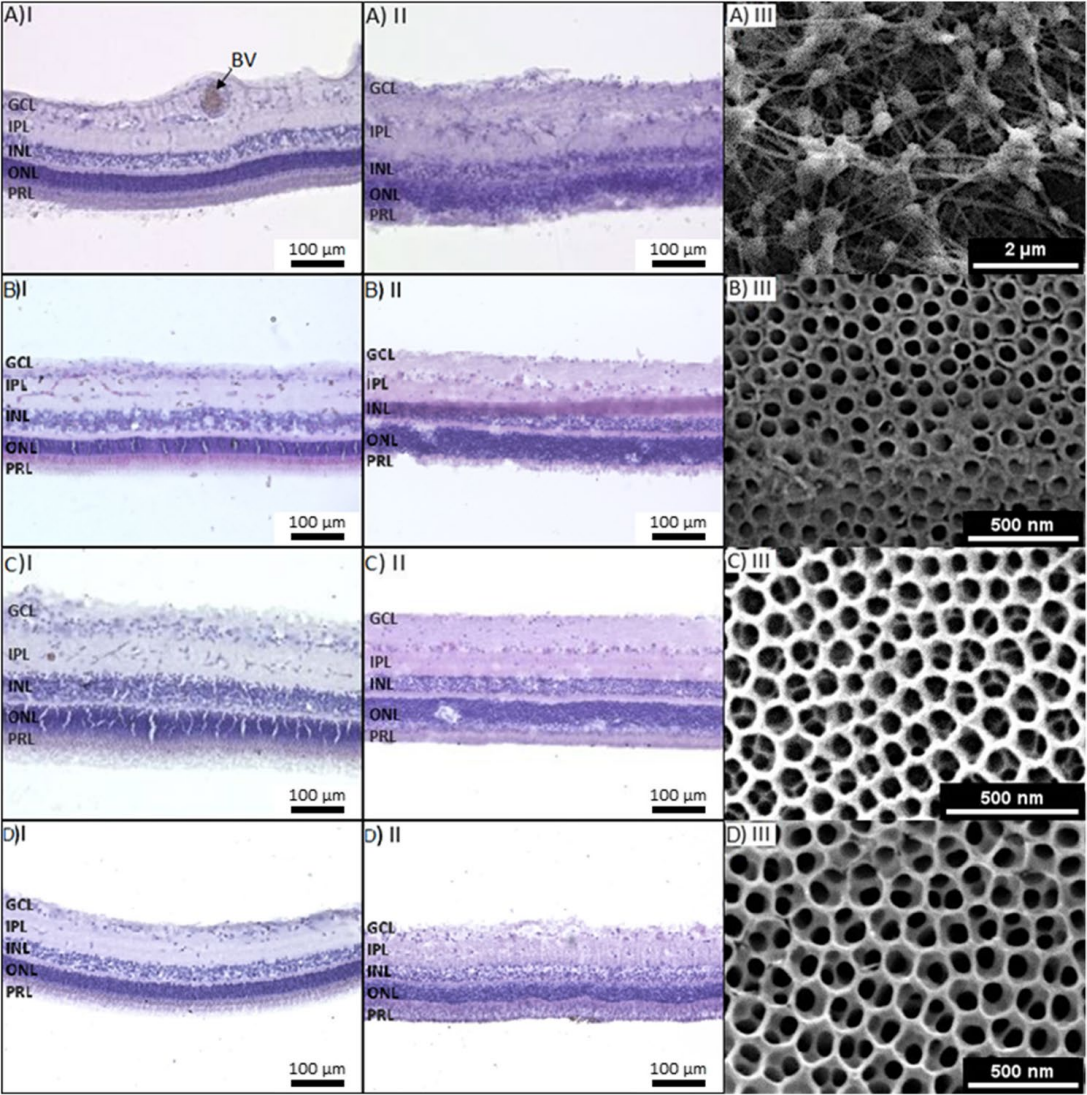
Organotypic culture of adult vascularized porcine retina explants on different scaffolds. Hematoxylin and eosin staining of a porcine retina before (I) and after (II) culture on different substrates for 5 days. Column (III) shows the corresponding cultivation substrate: **A** porcine retina cultured on Millicell^®^ filter, **B** single anodized nanotube scaffolds with tube diameters of 72 nm, **C** double anodized nanotube scaffolds with tube diameters of 72 nm and** D** double anodized nanotube scaffolds with tube diameters of 102 nm. After culturing, structure and tissue integrity are clearly visible for the double anodized nanotube substrates, but not for the Millicell^®^ filters or single anodized nanotube scaffolds. The layers of the retina are not maintained, indicating a loss of tissue integrity and properties of different layers. However, very good structural preservation (especially of the photoreceptor segments) was found on both double anodized nanotube scaffolds. BV: blood vessel, GCL: ganglion cell layer, INL: inner nuclear layer, IPL: inner plexiform layer, ONL: outer nuclear layer, PRL: photoreceptor segment layer

Nanotube Scaffolds (NTS) were synthesized by electrochemical anodization at room temperature. For this purpose, titanium foil (Advent Research Materials Ltd., 0.1 mm thickness, 99.6+ % purity) was cut into small pieces of 20 mm x 30 mm, cleaned with distilled water and isopropyl alcohol for 10 min each in an ultrasonic bath, and then dried in a nitrogen stream. The cleaned titanium foil was used as the anode and a platinum mesh as the cathode. Both electrodes were clamped in a holder with a fixed distance of 45 mm. The setup was immersed in an electrolyte (98% ethylene glycol, 2% distilled water) containing 0.3% or 0.6% ammonium fluoride for single or double anodization, respectively. For single anodized NTS, 0.3%-NH_4_F-electrolyte was used, and a voltage of 50 V was applied for 60 min, resulting in free-standing nanotubes with a tube diameter of 72 ± 3 nm. 0.6%-NH_4_F-electrolyte was used to synthesize double anodized NTS. The first anodization step takes 30 min at a voltage of 50–60 V [20]. At this production point, we produced single anodized nanotubes with a diameter of about 70–100 nm, respectively. The TiO_2_ nanotubes were then completely removed from the Ti-sheet by ultrasonication in distilled water followed by drying in a nitrogen stream. The pretreated Ti-sheet was subjected to a second anodization step for 30 min at a voltage of 50–60 V, resulting in a thin interconnected nanoporous surface layer a few nanometers thick, with underlying nanotubes. The tube diameter of the nanoporous layer is 72 ± 2 nm and 102 ± 5 nm, respectively. NTS were allowed to rest overnight in ethylene glycol. Finally, to remove any residual electrolyte, the scaffolds were cleaned with distilled water by ultrasonication for 10 min. Note: We tested additional tube diameters, single and double anodization, but for the sake of simplicity we have limited the description to the tubes shown in Fig. 1.

### Retina Preparation and Organotypic Culture of Porcine Retina

Adult retina from porcine eyes was kindly provided by the slaughterhouse Emil Färber GmbH Großschlächterei & Co. KG, Belgern Schildau, Germany. Pigs were slaughtered at the age of 6 to 7 months and weighted approximately 100 to 150 kg. After individual electrocution and subsequent exsanguination, pig eyes were enucleated by a qualified butcher, leaving 3–5 mm of the optic nerve intact. Eyes were transferred to ice-cold PBS (4–8 °C, pH 7.4) and transported to the laboratory. Transportation took about 2 h. The eyeball was cleaned of surrounding tissue such as muscle, fatty tissue and connective tissue, transferred to a tube filled with 70% ethanol for some seconds to sterilize the ocular surface, washed twice in sterile PBS. The eye was opened along the ora serrata under sterile conditions in warmed culture medium. After disconnecting the vitreous body, the retina was always punched square (13 mm x 13 mm) centrally below the optic nerve. The quadratic punched samples were always taken from the same origin or position of the eye, separated from their surroundings, detached from the retinal pigment epithelium and carefully placed with the photoreceptor side down on the NTS and on the Millicell^®^ cell culture inserts (hydrophilic PTFE-membrane, pore size 0.4 μm, PICM0RG50, Merck Millipore) for comparison purposes. Porcine retina was cultured using the air-liquid interface method as follows.

Therefore, NTS was placed on a sterile, stainless-steel grid (30 mm x 30 mm x 5 mm) inside a culture dish (Ø = 5 cm). The culture medium (Advanced DMEM/F-12, 0.1% gentamycin, 10% horse serum, 2% Glutamax) was added, so that the medium was in contact with the bottom of the scaffold but did not overflow it. This ensures a free gas exchange.

For cultures using the Millicell^®^ inserts, the retina was placed on an insert with the photoreceptor side down and inserted in a culture dish (Ø = 5 cm). Culture medium was added until the filter was wet, but not submerged. The cultures were incubated at 37 °C and 5% CO_2_ for 5 days.

Afterwards, the retina was fixed on top of the NTS or the filter with fixation solution (4% PFA in PBS, pH 7.4) for at least 4 h or overnight. Due to the strong adhesion of the tissue to the NTS, the tissue was carefully removed from the NTS surface with a razor blade. On the Millicell^®^ inserts the adhesion was weak, so they could be removed easily by a brush. The fixed tissues were then processed for histological staining (HE and immunohistochemical staining).

### Histological Processing (hematoxylin/eosin Staining, HE) and Brightfield Microscopy

30 μm slices of porcine retina of gradual *post mortem* times were stained with hematoxylin/eosin (HE) method to visualize tissue morphology from time point of enucleation at the slaughterhouse (*post mortem* hour 0) until *post mortem* hour 6. This experimental setting was chosen to simulate different transportation duration from slaughterhouse to bench, which we will call “*post mortem* time” in the following. During this time, the eyes were kept in ice-cool PBS solution. These time ranges were chosen to determine potential effects of *post mortem* ages on retinal tissue degradation and consequently on cultivation success any potential limit of *post mortem* preparation times. Therefore, the pig eyes were kept in ice-cool PBS from the time of enucleation until appropriate *post mortem* time and were then prepared as described above for NTS culture, but once the retina was isolated, it was not cultivated on NTS, but fixed in 4% PFA (in PBS, pH 7.4).

Fixed retinal samples were washed in PBS (pH 7.4) and embedded in 3% agarose. Samples were cut into 30 μm thin tissue slices using a vibratome and stored in PBS-NaN_3_ at 4 °C until further processing for staining. Slices for *post mortem* analysis were stained with hematoxylin and eosin (HE). Therefore, slices were briefly rinsed in PBS, transferred to distilled water and mounted on a microscope slide. The slides were then placed on a heating plate (35 °C) until evenly dried and fixed. After desalting the slides in a cuvette filled with distilled water, hematoxylin was applied for 4 min. Slides were then washed three times with water within 30 min, before eosin was applied for 4 min. The slides were then immersed in distilled water, then in 80% ethanol and finally in distilled water again until the agarose was clear. Excess water was removed, the slides dried on a heating plate and immediately covered with mounting medium on a coverslip. Slides were stored upright, at room temperature and protected from light for 24–48 h before microscopic analysis using a Keyence fluorescence microscope (BZ-9000, Keyence, Neu-Isenburg, Germany).

### Vital Dye Staining of Living Retinal Tissue Ex Vivo and Fluorescence Microscopy

**Fig. 2:**
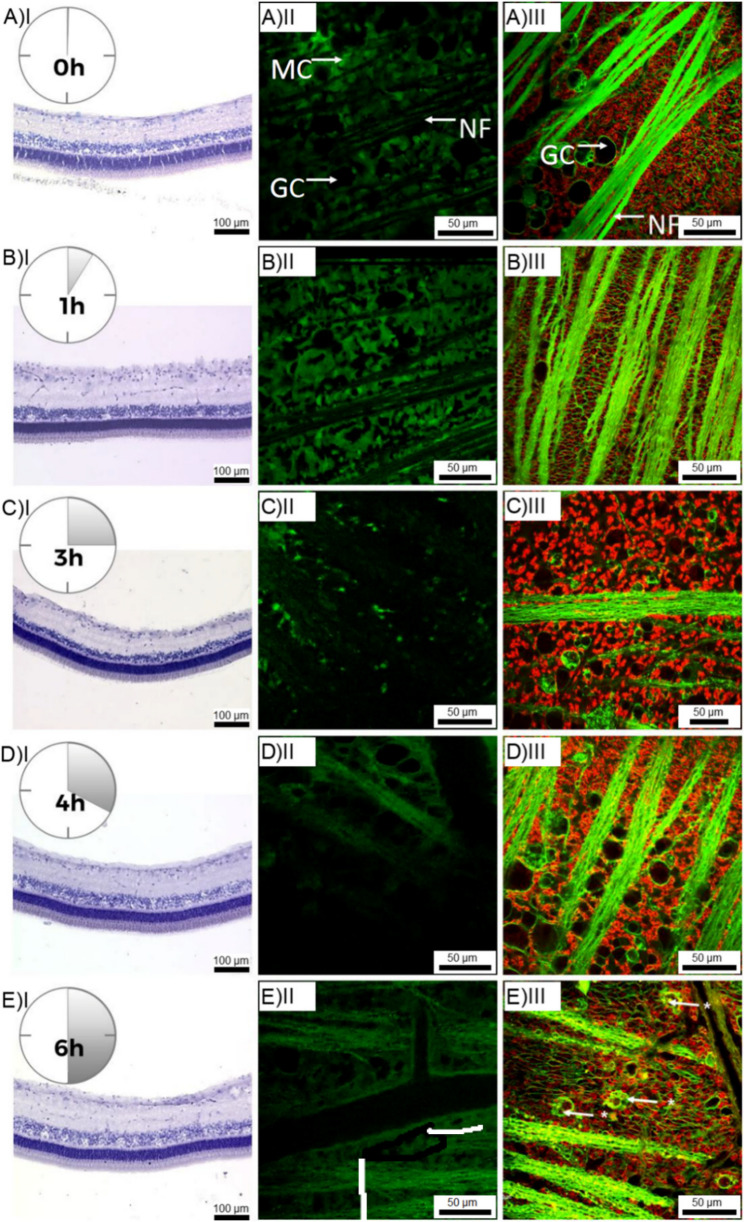
Morphology and viability of porcine retina at 0–6 h *post mortem*, symbolizing the effect of long transportation times from slaughter house to bench. Column I: hematoxylin/eosin staining of porcine retinas PFA-fixed at 0, 1, 3, 4 and 6 h *post mortem.* Column II: isolated living retinas incubated with in vivo fluorescent dye CellTracker Green (CTG), Müller glia cells were exclusively CTG positive and appear in green. Nerve fibers and ganglion cells somata (AII) appear black displaying no fluorescent staining. In E II a branched blood vessel is recognizable. Column III: retinas incubated with in vivo fluorescent dye MitoTracker Orange (MTO) and FM1-43 (green). MTO staines exclusively Müller cells and intact Müller cells/Müller cell end feet appear in orange. FM1-43 staines nerve fibers/ganglion cell axons and the cell membrane of ganglion cell somata. The distribution pattern of these in vivo fluorescent dyes are very specific and display the status of oxidative stress in the isolated retinal tissue. In E III (arrows) some ganglion cell somata were filled with FM1-43 showing the first damage of the integrity of the cell membrane due to oxidative stress. GC: ganglion cells, MC: Müller glial cells, NF: nerve fibers, BV: blood vessel

All steps were performed at room temperature. Living (non-PFA-fixed) retinal wholemounts were explanted and mechanically fixed on Whatman Filters with their vitread surface up, and placed into extracellular solution (136 mM NaCl, 3 mM KCl, 2 mM CaCl2, 1 MgCl2, 10 mM HEPES, 11 mM glucose), adjusted to pH 7.4. The wholemounts were loaded with vital dyes resolved in extracellular solution: 1 µM MitoTracker Orange (chloromethyltetramethylrosamine, M-7510, Invitrogen), CellTracker Green (5-chloromethylfluorescein diacetate; C-2925, Invitrogen) and FM™ 1–43 (Invitrogen). After appropriate incubation time, the wholemounts were transferred to a dye-free, extracellular solution filled chamber and examined using a confocal laser scanning microscope LSM 510 (Zeiss, Oberkochen, Germany) by viewing from the vitread side. The following cell membrane-permeant thiol-reactive vital dyes were tested: See Fig. 2, A-E; II and III.

### Immunohistological Staining and Fluorescence Microscopy

Porcine retinae was cultivated on two types of NT scaffolds (tube diameter 72 nm ± 3 nm and column 102 nm ± 5 nm) at 37 °C for 5 days. Retinae were fixed with 4%-PFA over night and carefully removed from NT scaffold. For immunohistological processing the tissue was embedded in 3% agarose (in PBS, pH 7.4). Samples were cut into 30 μm thin tissue slices using a vibratome and transferred into well-plates for staining process. The following steps were performed by using PBS (pH 7.4) + 1% DMSO + 3% triton-X-100, for incubation and washing. The slices were incubated in 5% donkey normal serum (Jackson Immunoresearch) for 1 h at room temperature and then incubated with primary antibody overnight at 4 °C (1:500 anti-GFAP by Dako; 1:100 anti-GαT1 by Santa Cruz; 1:100 anti-GαT2 by Santa Cruz, 1:500 anti-Glutmanine synthetase (GS) by Merck Millipore; 1:500 anti-IBA by Wako; 1:100 anti-PNA by Sigma; 1:500 anti-S100β by Sigma, 1:100 anti-Vimentin/V9 by Dako). After washing the slices 3 times for 1 h each, the fluorescent secondary antibodies (1:200, Jackson Immunoresearch) and Hoechst33358 (H3570, 1:1000, Life Technologies) were added and incubated for 2 h at room temperature and protected from light. Slices were washed, fixed and mounted on a microscope slide and stored upright until microscopic analysis using a confocal laser scanning microscope LSM 510 (Zeiss, Germany).

## Results

With the aim of developing a novel culture substrate for an organotypic long-term tissue preservation we present the most suitable surface parameters for a long-term culture of adult porcine retina using TiO_2_ nanotube scaffolds. We focused on adapting our technology to porcine tissues from slaughter houses and established a culture system that is not based on animal testing. It can also be used by researchers who cannot perform animal experiments for ethical, logistical, or cost reasons by demonstrating that animal tissue from factory farms (slaughter house) can be used in research.

The time frame in which the retina preparation could be performed was predetermined and set to a maximum of 4 h *post mortem*, as the degeneration of the retinal tissue had not yet advanced (see Fig. 2). We found little oxidative stress in the *post mortem* retinas at this *post mortem* age, Müller cell end feet formed a closed cell complex, which surrounded the ganglion cells without gaps. Past 4 h *post mortem*, the first irregularities became visible (altered cell volumes, presumably triggered by osmotic changes). In addition, the first ganglion cells filled with FM1-43 are visible, allowing the dye to penetrate through damaged cell membranes.

We continuously investigated and optimized different nanotube geometries and their influence on the porcine long-term culture. We compared tissue integrity of porcine retinae fixed on Millicell^®^ culture inserts (hydrophilic PTFE Millicell^®^ culture inserts, Merck) with those cultured on nanotube scaffolds, respectively. Retinae of eyes of different *post mortem* ages were used; the eyes were kept cold in buffer until the time of preparation (*post mortem*) and were closed during the entire *post mortem* time. They were opened for immediate preparation, dying and LSM analysis once *post mortem* hour was reached. TiO_2_ nanotube scaffolds were electrochemically anodized in an ammonium fluoride containing electrolyte. Detailed information is provided in the Materials and Methods section. Nanotube parameters were varied, including variation of tube diameter, single versus double anodized nanotube scaffolds, resulting in free-standing tubes and porous surfaces, respectively, or adjusting the composition of the culture medium. A certain combination of the parameters mentioned above led to culture success. We will present examples that showed best the systematically improved preservation of retinal morphologic integrity. We compared tissue morphology using hematoxylin and eosin (H&E) staining of retinas after 5 days in culture on Millicell^®^ inserts, single anodized nanotube scaffolds (72 nm ± 2 nm) and double anodized (72 nm ± 3 nm & 102 nm ± 5 nm) nanotube scaffolds. The morphology of the retina is shown in Fig. 1. The control staining (before culture) in **A**I) clearly showed the layered structure of the retina, whereas the morphology in the Millicell-cultured retina in **A**II) was severely impaired, less defined, swollen and the layered structure barely recognizable. This suggests that cell integrity is strongly altered, probably leading to neural damage and degeneration. Many studies suggest that adhesion has a positive influence on cell viability and structure maintenance and can be promoted by single cell or ECM attachments [19, 24, 25]. While we did not see a strong adhesion from tissue to the Millicell^®^ insert, we clearly saw it on the nanotube surface, and it became clearer with optimized nanotube parameters. Remaining tissue remnants have already been shown in another study by Friebe and Mayr [20].

Figure 1B shows the retinal structure before and after culture on single anodized nanotube scaffolds with a tube diameter of 72 nm. The retinal layers were still visible but showed less defined boundaries and appear rather bloated and swollen. Neuronal degeneration and damage are very likely. Figure 1C & **D** show the retinal structures on double anodized nanotube scaffolds, with tube diameters of 72 nm and 102 nm, respectively. The morphology was well preserved (**C**II & **D**III). The retinal layers are clearly distinguishable and correspond to the control slices, indicating that cell integrity was well maintained.

**Fig. 3:**
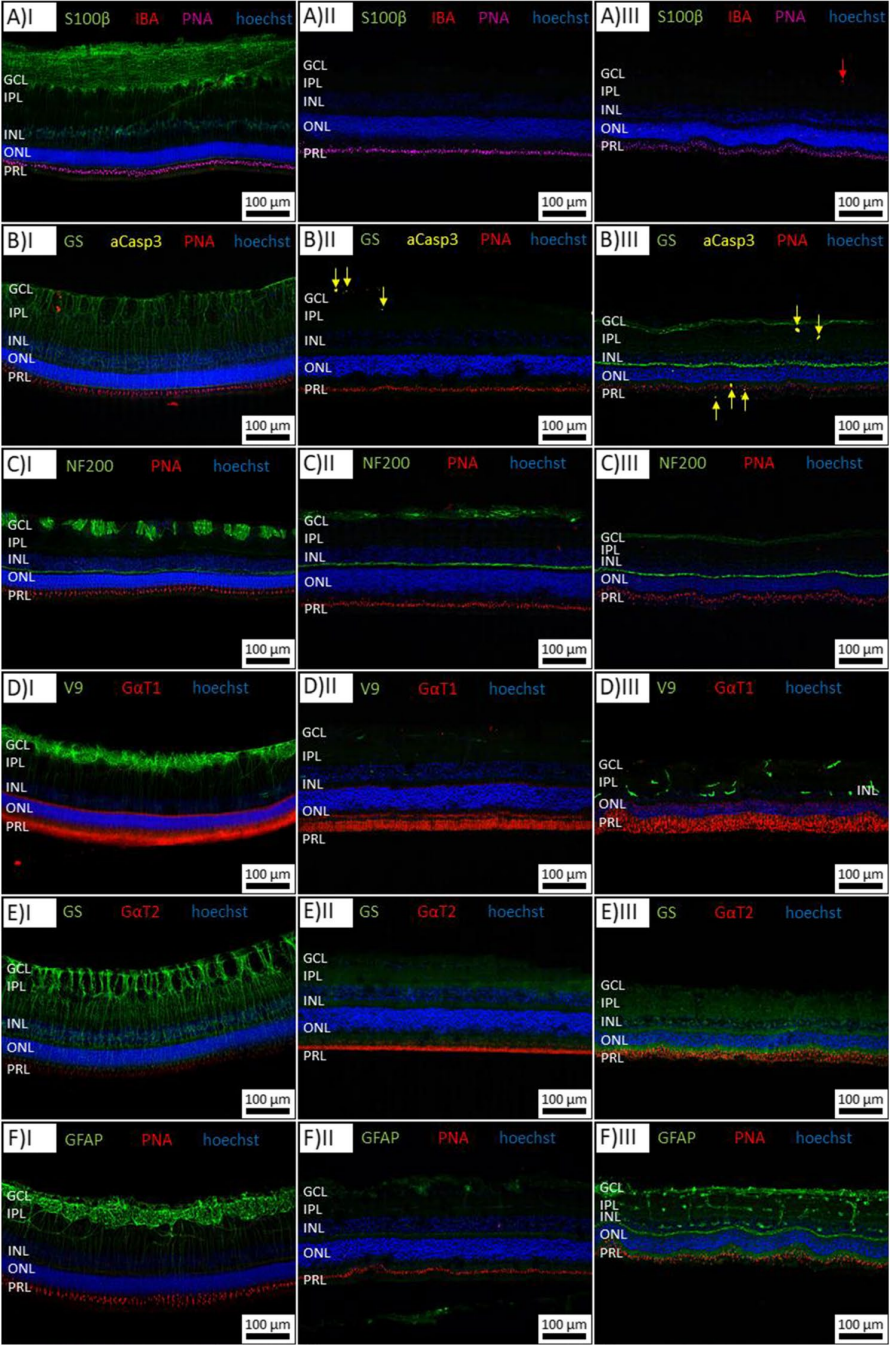
Immunofluorescent staining of porcine retina cultured on NT scaffolds of different characteristics: antibodies staining for specific retinal cellular markers whose distribution provides information about the histologic and biochemical integrity of the tissue, such as markers for photoreceptors, microglia, Müller glial cells, apoptosis, cell nuclei, neurons and others. Column I: porcine retina before culture (control), column II: porcine retina after 5 days cultivation on double anodized scaffolds with tube diameters of 72 nm ± 3 nm and column III: porcine retina after 5 days cultivation on double anodized scaffolds with tube diameters of 102 nm ± 5 nm. Please note: The retinal slices come from different cross-sectional planes (crosswise versus longitudinal) and appear differentially. As an example, retinal slices in row 3 come from different cross-sectional planes (crosswise CI and longitudinal CII and CIII) and the NF200 staining (neuro-filaments) appears differentially. However, the overall and cell specific staining pattern differ not essentially S100β: shows alterations in the ganglion cells glial cells/Müller cells, IBA: microglial marker, PNA: photoreceptor marker (mostly cones), Hoechst: cell nuclei staining, GS: glutamine synthetase in Müller cells, aCasp3: apoptosis marker, NF200: horizontal neurofilament/nerve fibers marker, V9: Vimentin marker (expressed by radial glial cells and astrocytes), GαT1: retinal rod segments, GαT2: retinal cone segments, GFAP: astrocytes and Müller glia cells. GCL: ganglion cell layer; IPL: inner plexiform layer; INL: inner nuclear layer; ONL: outer plexiform layer; PRL: photoreceptor segment layer

In addition, immunofluorescent analyses were performed for both double anodized nanotube scaffolds (72 nm and 102 nm tube diameter), as they showed the best tissue preservation (data were provided in the Supporting Information). We used antibodies that specifically bind cellular markers, whose distribution provides information about the histologic integrity of the tissue (including antibodies against photoreceptor cells, microglial cells, Müller glial cells, apoptosis, and cell nuclei). These preliminary results showed good histological preservation of photoreceptor cells and cell nuclei on both nanotube scaffolds diameter. The preservation of Müller glial cells and astrocytes was best on those nanotube scaffolds. In addition, we found indications for an inhibited glutamine synthetase for both scaffold types (data not shown). which could be influenced by the addition of hormones or glutamax (alternative to L-glutamine with higher stability) [26]. Microglia activation was only detected in a few cells. A general microglial reactivity could not be observed. Detailed results of the immunohistological stainings are presented in Fig. 3. All specific cellular markers show distinct and normal morphological appearance, no signs of glial reactivity of microglial cells (no increase of IBA staining) and/or macroglial cells (no increase of GFAP and S100β expression in astrocytes or Müller cells). Although, a decrease of GS staining in glial cells could be detected. Furthermore, rare detection of apoptotic cells (aCasp3), well preserved arrangement of photoreceptor segments (GαT1 and GαT2) and nuclear cell layers of intraretinal neuronal cells (Hoechst). Nerve fibers show well preserved arrangement.

Besides, the nanotube surface was examined by an environmental scanning electron microscope (ESEM) to investigate possible tissue residues after porcine retina culture. An adhesive border forms between the tissue and the scaffold, especially along the edges of the tissue, but also at regular intervals under the tissue (typical tissue remnants see Supporting Information - Fig. S1). Other nanotube scaffolds (e.g. single anodized) demonstrated weak retinal adhesion. Here ESEM showed tissue culture remnants, composed of many single cells, which might have migrated out of the tissue.

## Discussion

The aim of this study was to investigate the influence of different structured nanotube scaffolds on the tissue integrity and structural preservation of adult porcine retina in an ex vivo culture system. Systematically, the effect of the tube diameter and surface morphology (single and double anodized) on the success of long-term organotypic culture of the retina was tested. These results were compared to the culture on Millicell^®^ inserts. Of course, there are examples of successful culture on the Millicell^®^ inserts, especially for embryonic tissue or stem cells, but they do not allow organotypic long-term culture, for example of a sensitive, vascularized, adult and complex tissue such as porcine retina [21, 27, 28]. In our study, morphology preservation and culture time on Millicell^®^ inserts were insufficient for further use for scientific or pharmacological purposes. The retinal structure could only be preserved for a maximum of 3 days or less. The retina adheres poorly to the insert or swims loosely on top, and although the supply of nutrients and other important substances is ensured, degeneration is clearly advanced after a few days. However, proper adhesion of the tissue to the substrate can be crucial for cell and tissue survival during culture [29, 30]. With our adjustable nanotube scaffold culture system tissue adhesion was achieved. Here, an adhesive border was found. The double-anodized nanotube scaffolds (tube diameter of 72 nm and 102 nm) preserved the retinal morphology over 5 days. Thus, they support adhesion requirements of the porcine retina very well. Also, preliminary immunofluorescent analyses indicate best retinal preservation on the 72 nm and 102 nm diameter nanotube scaffolds. These analyses need to be expanded and validated for a meaningful result. However, the single-anodized nanotubes cannot sufficiently maintain the structure and tissue integrity, another indication of individual tissue requirements.

However, double anodized nanotube scaffolds are made of two structures, a thinner connected nano-porous layer on top with a thicker layer of underlying vertical aligned nanotubes. During anodization, the nanotube structure forms self-assembled [31, 32]. Those vertical aligned nanotubes have a slightly smaller tube diameter than the nano-porous structure above and probably promote the consistent nutrient and water supply. In our study, the photoreceptor outer segments connected to the nanotube surface. In natural environment, they were in contact to the microvilli network of the retinal pigment epithelium, which is a monolayer of 10–20 μm sized cells in hexagonal shape when viewed from above [33–35]. We believe that the tailorable and modifiable surface properties, the natural biocompatibility, and the superhydrophilic properties due to the surface structure enable successful organotypic tissue preservation. The hexagonally shaped nanotopography must match a typical range of adhesion points of retinal single cells (i.e. photoreceptor segments and microvilli) or ECM molecules [18]. 

Also, the air-liquid interface culture system was chosen based on its proven benefits for organotypic retinal explants. This setup ensures optimal oxygenation, prevents tissue swelling, and maintains the laminar structure of the retina over several days, as confirmed by our results and previous studies [23, 29]. While the lack of vascular supply and the exclusive diffusion of nutrients from the basal side represent limitations, the air-liquid interface method remains the gold standard for ex vivo retinal culture. Future studies could systematically compare air-liquid interface and fully submerged cultures to further optimize nutrient supply and tissue preservation, especially for extended culture periods.

Previous studies have shown that an avascular adult guinea pig retina can be maintained for several weeks [19]. In the current experiments with porcine retina, the maximum cultivation time, with simultaneous structural maintenance has not been exhausted. Also, investigations of the morphology preservation can be supplemented, for instance, by gene expression experiments.

Differences in immunostaining, e.g. for GS, NF200, and GFAP between the two scaffold types, suggest that the underlying substrate can influence retinal cell health and stress responses. As can be seen from Fig. 3 (column III), reduced GS and increased GFAP expression are indicative of glial activation and metabolic stress in Müller cells, while changes in NF200 staining may reflect differences in neuronal microenvironment of the cultured retina, affecting both glial and neuronal populations. Our previous and current results show the possibility to tailor the nanotube surface to individual tissue requirements due to their customizable surface characteristics [18, 19]. The fact that the sensitive porcine retina survives on the hard nanotube surface cannot yet be fully explained but Taylor et al. shows that tissue survival is strongly influenced by lateral applied tensions in a long-term culture of porcine retina [36]. An applied force as well as a generated adherence to a surface, result in a direct interaction between the tissue and the substrate and thus to an increased tissue and cell survival. Recent advances in retinal tissue culture have introduced a variety of biomimetic scaffolds including hydrogels, electrospun nanofibers, and 3D-printed matrices composed of bioactive and biodegradable polymers. These systems aim to mimic the extracellular matrix, support cell survival, and in some cases, allow co-culture with the RPE to more closely replicate in vivo conditions [37–40]. The nanotube scaffold developed in our study offer several notable advantages. The surface geometry can be precisely tunes to optimize cell and tissue adhesion, mechanical support, and nutrient exchange, supporting the preservation of adult, vascularized retina explants [20, 22, 23]. There are robust and chemically inert, providing a stable and reproducible substrate for long-term culture without the risk of rapid degradation or swelling [20, 22, 23].The nanotube surface enables a reliable surface attachment of the retina, even under mechanical stress, without additional adhesives [23]. Furthermore, the scaffolds can be regenerated and reused after long-term culture, reducing cost and material waste [20]. The nanotube surface enables a reliable surface attachment of the retina, even under mechanical stress, without additional adhesives [23]. Furthermore, the scaffolds can be regenerated and reused after long-term culture, reducing cost and material waste [20]. Nevertheless, our study challenging some important limitations at this stage of development, like the lack of active vascular perfusion, limiting nutrient and oxygen supply to diffusion only, which may affect the viability of inner retinal layers and limited biochemical complexity compared to native retinal tissue, as is it also the case for most current scaffolds systems [37–40]. Our systems lack the integration of the RPE, which mimics more the in vivo retinal environment [38, 40]. The limited vascular immunostaining in this study restricts conclusions about blood vessel viability and integrity during culture. Immunohistochemical differences between scaffold types (e.g. single anodized versus double anodized NTS) suggest substrate-dependent effects on glial and neuronal health, indicating a need for further optimization of nanotube geometry or surface chemistry.

## Conclusions

In conclusion, literature lacks numerous meaningful studies on long-term culture of tissue slices. With the excellent properties of the nanotube scaffolds, organotypic culture of adult porcine retina could be enabled and improved. This has never been done before and is reported here for the first time. Our culture system finds possible medical application in basic or preclinical research since the porcine retina closely resembles the human retina. In perspective, diseases and drug responses can be better understood than in commonly used mouse models. Thus, this approach can contribute to the reduction of animal testing since the porcine eyes were obtained from the slaughterhouse and increase the transferability of the results to humans, as it might be used for other animal or human tissues.

## Supplementary Information

Supplementary Material 1.

## Data Availability

Data and materials availability: All data are available in the main text or the Supporting Information.
