# Supplementary material for: Organotypic Culture of Adult Vascularized Porcine Retina Explants In Vitro on Nanotube Scaffolds
Source: Biol Proced Online. 2025 Sep 8;27:35. doi: 10.1186/s12575-025-00301-5 (PMC12418674; doi:10.1186/s12575-025-00301-5)
Supplement: Supplementary file 1 — Supplementary Material 1. [file 12575_2025_301_MOESM1_ESM.pdf]

Supporting Information

Organotypic Culture of Vascularized Adult Porcine Retina Explants *in vitro* on Nanotube Scaffolds

Authors: Sabrina Friebe[1,2,3]†, Solveig Weigel[4]†, Mike Francke[4,5], Stefan G. Mayr[1,2]*

Affiliations:

[1]Division of Surface Physics, Department of Physics and Earth System Sciences, University of Leipzig, Linnéstr. 5, 04103 Leipzig, Germany.

[2]Department of Biocompatible and Bioactive Surfaces, Leibniz Institute of Surface Engineering (IOM), Permoserstr. 15, 04318 Leipzig, Germany.

[3]current address: Medical Informatics Center, University Hospital Leipzig, Härtelstr. 16-18, 04107 Leipzig, Germany.

[4]Paul-Flechsig-Institute of Brain Research, University of Leipzig, Liebigstr. 19, 04103 Leipzig, Germany.

[5]current address: University Clinic and Polyclinic for Ophthalmology, University Hospital Halle (Saale), Ernst-Grube-Str. 40, 06120 Halle (Saale), Germany.

*Corresponding author: Prof. Dr. Stefan G. Mayr, Division of Surface Physics, Department of Physics and Earth System Science, University of Leipzig, Linnéstr. 5, 04103 Leipzig, Germany, Email: smayr@uni-leipzig.de.

†These authors contributed equally to this work

The PDF file includes:

Fig. S1
Fig. S2
Fig. S3
Tab. S1

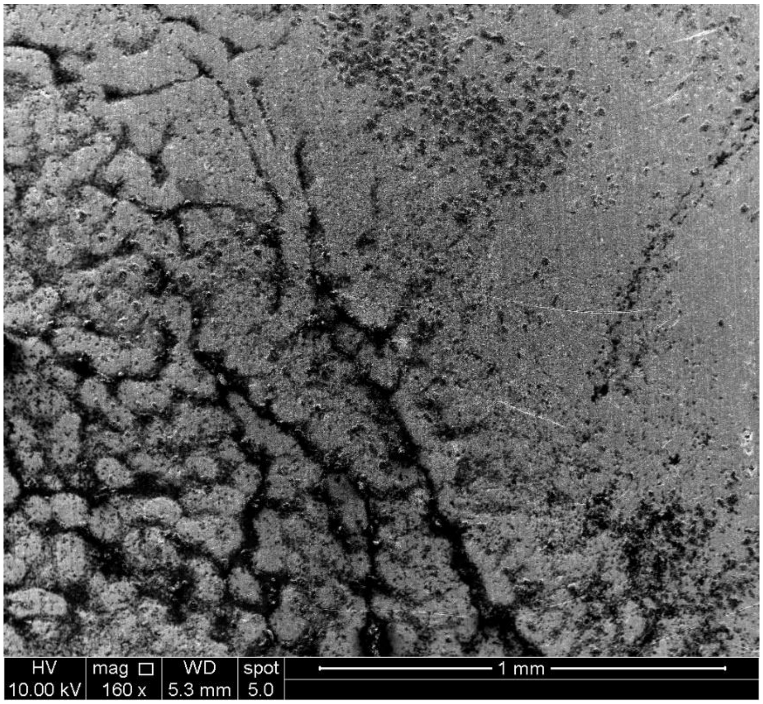

Fig. S1: The SEM image shows the tissue remnants of porcine retina after 5 days in culture on nanotube scaffolds with a tube diameter of 100 nm. Areas with many tissue remnants adhered very strongly to the nanotube surface. Areas with fewer remnants probably allowed for nutrient supply.

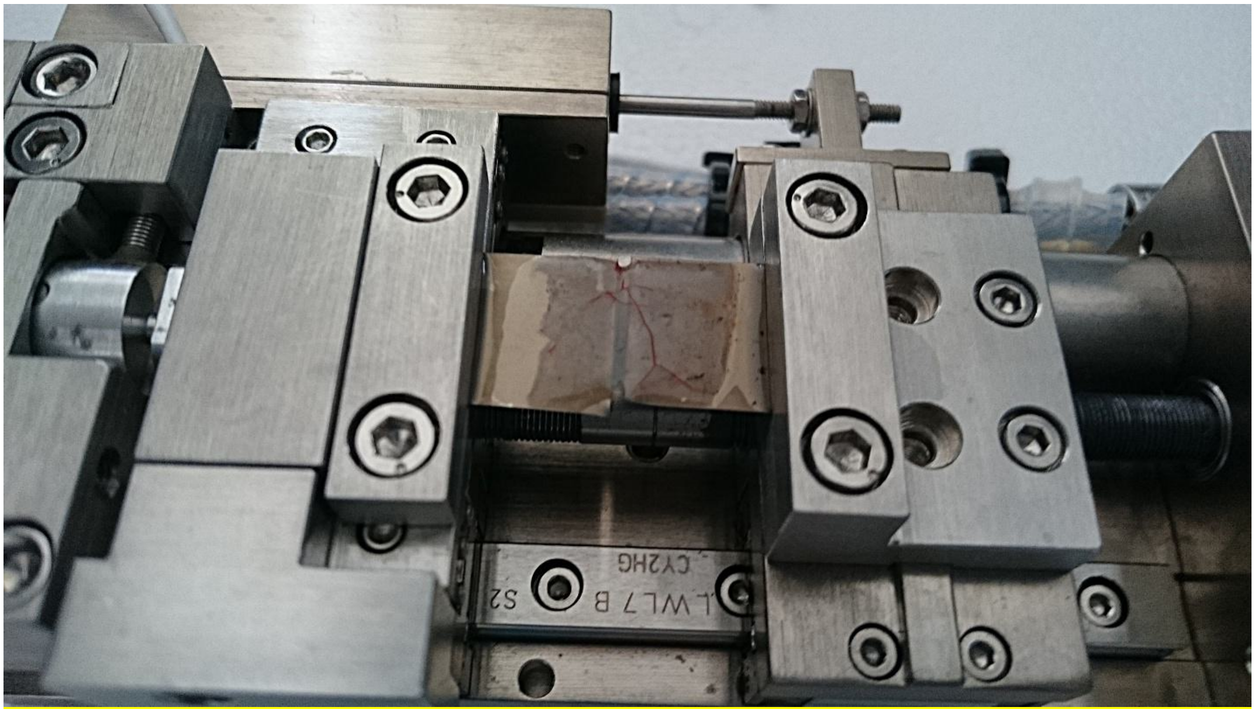

Fig. S2: The image shows an adherent adult porcine retina (cultured for 24h) on top of two separate, parallel aligned nanotube scaffolds clamped inside a tensile stage (DEBEN Microtest) to measure its mechanical properties.

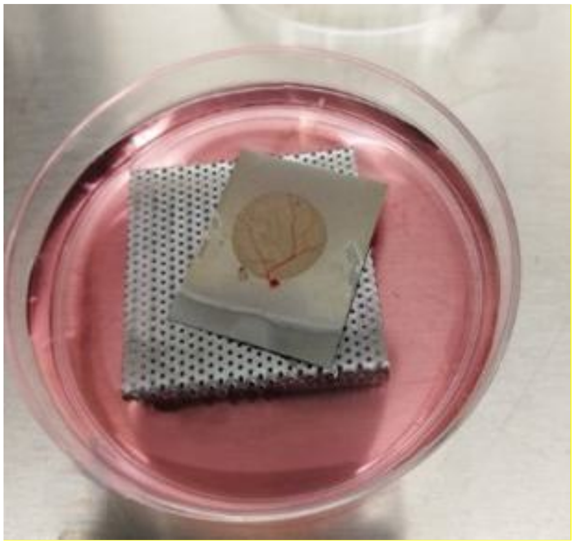

Fig. S3: The image shows the culture setup in top view. The (round-shaped) porcine retina explant is placed on a nanotube scaffold, which lays on a stainless-steel grid inside a petri dish. The culture medium is filled up to the edge of the nanotube scaffold, but does not submerge it. Due to the super-hydrophilic properties of the nanotube scaffoldy, the medium is transported over it, creating a thin layer of culture medium, supplying nutrients to the retinal tissue. Oxygen supply to the inner retina tissue might be supplied from the air-liquid interface.

**Culture parameter**		**Nanotube scaffold parameter**					
		**Tube diameter [nm] single anodized**			**Tube diameter [nm], double anodized**		
Medium	Serum concentration [%]	50	70	100	50	70	100
AMES	10	×	×	×			
	5	×	×	×			
DMEM F12 advanced	10	×	(✓)	(✓)		✓	✓
	6		×				
	5	×	×	×		(✓)	x
	4		×				
	2.5		×			×	
	0		×			×	
	d1: 0→d2: 2.5		×				
	d1: 0→ d2: 5		×			×	×
	d1: 0→ d2: 10		×			×	(✓)

Tab. S1: The table shows the different investigated combinations of medium and nanotube **parameter** diameter and culture success. Empty field: not tested; ✓ YES , (✓) partially, × NO, **double** anodized NT > 100 nm due to NTS instability
